# Up-regulated IL-36 expression promotes a pro-tumorigenic phenotype in pancreatic ductal adenocarcinoma

**DOI:** 10.1016/j.gendis.2025.101973

**Published:** 2025-12-09

**Authors:** Jianhong An, Quanxing Liao, Ziqin Yu, Zihao Du, Juan Du, Qiang Xiao, Tingting Jiang, Changwen Huang, Keping Xie

**Affiliations:** a Department of General Surgery, The Affiliated Qingyuan Hospital (Qingyuan People's Hospital), Guangzhou Medical University, Qingyuan, Guangdong 511518, China; b Center for Pancreatic Cancer Research, South China University of Technology, Guangzhou, Guangdong 510006, China

Pancreatic ductal adenocarcinoma (PDAC) is one of the most aggressive and lethal solid tumors, ranking as the fourth leading cause of cancer-related mortality worldwide. Despite advances in therapeutic strategies, the five-year survival rate remains less than 10%.^1^ This poor prognosis stems primarily from late diagnosis, intrinsic resistance to adjuvant therapy, and a profoundly immunosuppressive tumor microenvironment. Chronic inflammation is well recognized as a major risk factor and driver of PDAC tumorigenesis, with pancreatitis representing a significant precursor lesion.^2^ Among the proinflammatory mediators involved, interleukin (IL)-1 family cytokines have garnered attention due to their central role in immune modulation, tumor cell survival, and response to treatment.

IL-36 cytokines, including IL-36α, IL-36β, and IL-36γ, are recently characterized members of the IL-1 family that signal through the IL-36 receptor (IL-36R), a complex composed of IL-1 receptor-related protein 2 (IL-1Rrp2) and the IL-1 receptor accessory protein (IL-1RAcP). This signaling axis is tightly regulated by the natural antagonist IL-36 receptor antagonist (IL-36RA) and, more broadly, by IL-38, another IL-1 family member with anti-inflammatory properties.^3^ While originally identified in epithelial barrier tissues such as the skin and lungs, IL-36 cytokines have since been implicated in a broad spectrum of inflammatory conditions, including psoriasis, inflammatory bowel disease, and chronic pulmonary disorders.

Recent research has uncovered context-dependent roles for IL-36 signaling in cancer. In some settings, IL-36 enhances anti-tumor immunity by promoting CD8^+^ T cell activation and the formation of tertiary lymphoid structures, contributing to tumor control.^4^ However, in other malignancies, such as gastric, lung, and colon cancers, IL-36 signaling has been associated with pro-tumorigenic effects, including increased tumor cell proliferation, migration, immune evasion, and poor prognosis.^5^ Despite the emerging relevance of IL-36 in cancer biology, its specific function in pancreatic cancer remains undefined. In the present study, we investigated the expression and functional role of IL-36 signaling in PDAC through clinical tissue analysis, *in vivo* tumor models, and mechanistic validation.

Immunohistochemical analysis of human pancreatic tissue microarrays revealed significant up-regulation of IL-36α, IL-36β, IL-36γ, and IL-36R in PDAC specimens and samples from patients with chronic pancreatitis compared with normal pancreatic tissues (Fig. 1A). Notably, both IL-36β and IL-36R were moderately elevated in chronic pancreatitis tissues relative to normal controls, indicating that IL-36 signaling activation may occur early in the inflammatory cascade preceding malignant transformation. These findings suggest that the IL-36 axis may be involved in the chronic inflammatory environment that contributes to PDAC pathogenesis.

**Figure 1:**
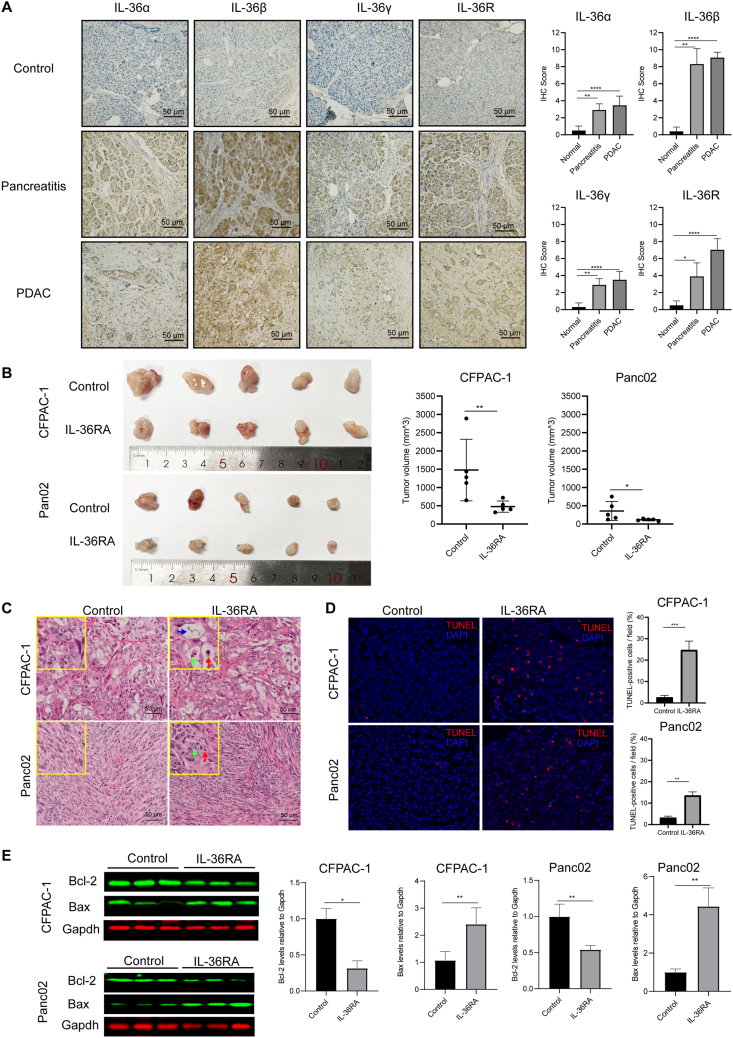
IL-36 signaling promotes a pro-tumorigenic phenotype in PDAC. **(A)** Representative immunohistochemical staining of IL-36α, IL-36β, IL-36γ, and IL-36R in human normal pancreas, chronic pancreatitis, and PDAC tissues. Immunohistochemistry staining scores of IL-36α, IL-36β, IL-36γ, and IL-36R were evaluated in normal pancreatic tissue (*n* = 10), chronic pancreatitis tissue (*n* = 10), and PDAC tissue (*n* = 30). Immunohistochemistry scores were calculated by multiplying staining intensity (0–3) and the percentage of positive cells (0–4), yielding a total score ranging from 0 to 12. Bars represent mean ± standard deviation. Statistical significance was determined using the Kruskal–Wallis test followed by Dunn's post hoc test for pairwise comparisons between normal and disease groups. ∗*P* < 0.05, ∗∗*P* < 0.01, and ∗∗∗*P* < 0.0001. **(B)** Representative images of excised tumors and quantification of end-point tumor volumes from CFPAC-1 xenografts in nude mice and Panc02 tumors in C57BL/6 mice treated with IL-36RA or vehicle control. *n* = 5 mice per group. ∗*P* < 0.05 and ∗∗*P* < 0.01. **(C)** Hematoxylin and eosin staining of tumor sections showing increased apoptotic morphology in IL-36RA-treated tumors compared with controls. Nuclear pyknosis (red arrows), karyorrhexis (green arrows), and karyolysis (blue arrows) are indicated. **(D)** TUNEL staining of tumor sections showing increased apoptotic cells in IL-36RA-treated groups. Quantification of TUNEL-positive cells per field is presented on the right. ∗∗∗*P* < 0.001 and ∗∗*P* < 0.01. **(E)** Western blotting analysis of apoptosis-related proteins in CFPAC-1 and Panc02 tumors. GAPDH was used as a loading control. Quantification of relative protein levels was shown on the right. Data are presented as mean ± standard error of the mean from three independent experiments. ∗*P* < 0.05.

To further elucidate the role of IL-36 signaling in PDAC progression, we employed xenograft models using both human (CFPAC-1) and murine (Panc02) pancreatic cancer cell lines. Tumor-bearing mice were administered intraperitoneally with recombinant IL-36RA or vehicle control, and tumor growth was monitored over time. In both models, IL-36RA treatment led to a reduction in tumor volume as compared with controls, demonstrating a conserved tumor-promoting function for IL-36 signaling in distinct mouse models (Fig. 1B; Fig. S1A; Table S1). Histopathological examination showed hallmark features of apoptosis in the tumors from IL-36RA-treated mice, including nuclear pyknosis, karyorrhexis, and karyolysis (Fig. 1C). Consistently, TUNEL staining revealed a significantly higher proportion of apoptotic cells in the tumors from IL-36RA-treated mice, confirming enhanced apoptosis following IL-36 signaling blockade (Fig. 1D).

To further delineate the effects of IL-36RA on tumor cell behavior, we first examined its impact on pancreatic cancer cell viability *in vitro*. Treatment with IL-36RA alone did not significantly alter the viability of CFPAC-1 or Panc02 cells, consistent with the notion that basal IL-36 signaling is relatively weak in those cell lines. However, when cells were pre-stimulated with recombinant IL-36γ, subsequent IL-36RA treatment led to a marked reduction in cell viability, as measured by CCK-8 assays (Fig. S1B). These findings suggest that IL-36RA suppresses tumor cell survival preferentially under conditions of IL-36 pathway activation. In addition, IL-36RA did not alter PD-L1 expression in either CFPAC-1 or Panc02 cells under our experimental conditions (Fig. S1C).

To dissect the underlying molecular mechanism, we analyzed the expression of apoptosis-related proteins in tumor lysates by Western blotting. Treatment with IL-36RA led to marked down-regulation of the anti-apoptotic protein Bcl-2 and concurrent up-regulation of the pro-apoptotic protein Bax in both CFPAC-1 and Panc02 tumors. This shift in the Bcl-2/Bax ratio suggests that IL-36 signaling may favor tumor cell survival by suppressing mitochondrial apoptosis pathways and that IL-36RA reverses this effect by reactivating programmed cell death (Fig. 1E).

To better understand the clinical relevance of these findings, we examined data from The Cancer Genome Atlas (TCGA) PDAC cohort. Correlative analysis revealed that IL1RL2 (IL-36R) expression was positively associated with tumor-related gene signatures, including those linked to NF-κB pathway activation, inflammatory cytokine production, and anti-apoptotic signaling (Fig. S2). These bioinformatic results are consistent with our *in vivo* observations and reinforce the hypothesis that IL-36R acts as a functional mediator of tumor-promoting inflammation in PDAC.

Given the established link between pancreatic inflammation and tumorigenesis, we further explored the role of IL-36 in acute pancreatitis. Immunofluorescence staining demonstrated increased expression of IL-36α, IL-36β, IL-36γ, and IL-36R in cerulein-induced pancreatitis (Fig. S3A). Importantly, IL-36RA treatment markedly attenuated pancreatic injury as determined by hematoxylin and eosin staining, *e.g.*, preserved acinar architecture and reduced inflammatory infiltration compared with that from those treated with cerulein alone (Fig. S3B). Multicolor immunofluorescence further revealed decreased infiltrations of CD3^+^ T cells and F4/80^+^ macrophages in the IL-36RA group after 36 h of treatment, consistent with a dampened inflammatory response (Fig. S3C). These data suggest that IL-36 not only contributes to tumor maintenance but may also exacerbate inflammatory tissue damage in early stages of pancreatic disease, further supporting its role as a critical mediator connecting chronic inflammation and carcinogenesis and progression.

In summary, our study identifies IL-36 signaling as a previously underappreciated driver of pancreatic pathology. IL-36 and its receptor were up-regulated in PDAC and chronic pancreatitis, suggesting that IL-36 activation emerges early during pancreatic inflammation and persists through malignant transformation. Blockade of IL-36 with IL-36RA suppressed tumor growth in both immunodeficient and immunocompetent mouse models, highlighting a conserved pro-tumorigenic role of IL-36. Mechanistically, IL-36RA enhanced tumor cell apoptosis, as shown by increased TUNEL positivity and modulation of Bcl-2/Bax expression. *In vitro*, CCK-8 assays further revealed that the inhibitory effects of IL-36RA were evident mainly under conditions of IL-36γ stimulation, supporting a cell-intrinsic pro-survival role of IL-36 signaling. However, IL-36RA did not alter PD-L1 expression, and its anti-tumor effect is independent of immune checkpoint regulation.

Significantly, our findings extend to inflammatory disease beyond cancer. In acute pancreatitis, IL-36RA preserves acinar structure and reduces infiltration of CD3^+^ T cells and F4/80^+^ macrophages, indicating that IL-36 contributes to tissue injury by amplifying inflammatory cell recruitment, whereas IL-36RA attenuates inflammation. Therefore, our findings support a dual role for IL-36 in pancreatic diseases: promoting tumor cell survival in PDAC and exacerbating immune-mediated damage in pancreatitis.

However, several limitations should be noted in our current study. First, the use of immune-deficient models precludes assessment of the impact of IL-36 on anti-tumor immunity. Future studies employing immune-competent models combined with single-cell transcriptomic approaches would be logical to delineate the contributions of specific stromal and immune populations in inflammation and tumorigenesis. Second, while our work primarily focused on apoptosis, IL-36 may also regulate other hallmarks of cancer, including metabolic reprogramming and epithelial–mesenchymal transition, which warrant further investigation. Finally, we did not directly evaluate canonical downstream pathways, including MAPK and NF-κB, while future mechanistic studies are needed to clarify their functional involvement.

Collectively, our study nominates IL-36R as a promising therapeutic target in pancreatic pathology. Blocking IL-36 signaling suppresses tumor cell survival through enhanced apoptosis, while also mitigating inflammation-driven pancreatic injury. These findings suggest that IL-36 pathway inhibition may represent a novel therapeutic strategy in the management of pancreatic diseases, including PDAC and pancreatitis.

## CRediT authorship contribution statement

**Jianhong An:** Validation, Resources, Methodology, Investigation, Funding acquisition, Formal analysis, Data curation, Conceptualization. **Quanxing Liao:** Formal analysis, Data curation. **Ziqin Yu:** Data curation. **Zihao Du:** Data curation. **Juan Du:** Data curation. **Qiang Xiao:** Data curation. **Tingting Jiang:** Writing – review & editing, Supervision, Resources. **Changwen Huang:** Writing – review & editing, Supervision, Resources, Funding acquisition. **Keping Xie:** Writing – review & editing, Writing – original draft, Supervision, Resources, Project administration, Formal analysis, Conceptualization.

## Ethics declaration

Animal experiments were conducted in accordance with institutional guidelines and approved by the Animal Ethics Committee of Qingyuan People's Hospital (Approval No. LEAC-2023-013).

## Funding

This work was supported in part by the Scientific Research Project of Qingyuan People's Hospital (Guangdong, China) (No. 11018 to J.A.); and by the 10.13039/501100021171Guangdong Basic and Applied Basic Research Foundation (China) (No. 2023A1515111002 to J.A.), the 10.13039/501100001809National Natural Science Foundation of China (No. 82072632 to K.X.), and the Guangzhou Municipality Bureau of Science and Technology (No. 202102010033 to K.X.), Guangdong, China (No. 202102010033 to K.X.).

## Conflict of interests

Keping Xie is the member of *Genes & Diseases* Editorial Board. To minimize bias, he/she was excluded from all editorial decision-making processes related to the acceptance of this article for publication. The remaining authors declare no other conflict of interests.

## References

[1] Siegel R.L., Kratzer T.B., Giaquinto A.N., Sung H., Jemal A. (2025). Cancer statistics. CA Cancer J Clin.

[2] Shadhu K., Xi C. (2019). Inflammation and pancreatic cancer: an updated review. Saudi J Gastroenterol.

[3] Finucane M., Brint E., Houston A. (2025). The complex roles of IL-36 and IL-38 in cancer: friends or foes?. Oncogene.

[4] Zhao X., Chen X., Shen X. (2019). IL-36β promotes CD8^+^ T cell activation and antitumor immune responses by activating mTORC1. Front Immunol.

[5] Elias M., Zhao S., Le H.T. (2021). IL-36 in chronic inflammation and fibrosis - bridging the gap?. J Clin Investig.

