# Supplementary material for: Up-regulated IL-36 expression promotes a pro-tumorigenic phenotype in pancreatic ductal adenocarcinoma
Source: Genes Dis. 2025 Dec 9;13(4):101973. doi: 10.1016/j.gendis.2025.101973 (PMC13015218; doi:10.1016/j.gendis.2025.101973)
Supplement: Multimedia component 1 [file mmc1.pdf]

Upregulated IL-36 Expression Promotes a Pro-tumorigenic Phenotype in Pancreatic Ductal Adenocarcinoma

Jianhong An[1,2], Quanxing Liao[1], Ziqin Yu[1], Zihao Du[1], Juan Du[1], Qiang Xiao[1], Tingting Jiang[2], Changwen Huang[1*], Keping Xie[2*]

[1]Department of General Surgery, The Affiliated Qingyuan Hospital (Qingyuan People's Hospital), Guangzhou Medical University, Qingyuan, Guangdong 511518, China

[2]Center for Pancreatic Cancer Research, South China University of Technology, Guangzhou, Guangdong 510006, China

[*]Correspondence: mcxiekeping@scut.edu.cn (K.X.)；ncdxhcw@163.com (C.H.).

SUPPLEMENTARY INFORMATION

1. **Materials and Methods**
2. **Supplementary Tables**
3. **Supplementary Figures**

1. Materials and Methods

Tissue samples and immunohistochemistry

A commercially available human pancreas tissue microarrays (TMAs) were obtained from Bitiantech Co., Ltd. (DPA1004, Guangzhou, China). This array includes a total of 50 tissue samples, consisting of 30 cases of pancreatic ductal adenocarcinoma (PDAC), 10 cases of chronic pancreatitis, and 10 samples of histologically normal pancreatic tissue. Each case was represented by duplicate cores to ensure sample reliability and reproducibility. All samples were histopathologically confirmed, and relevant clinical information was provided by the manufacturer. The TMA was used for immunohistochemical analysis as part of the study to evaluate marker expression in various pancreatic conditions. TMAs were sectioned at 4 μm, deparaffinized, rehydrated, and subjected to heat-mediated antigen retrieval using Tris-EDTA buffer (pH 9.0). Sections were blocked and incubated overnight at 4 °C with primary antibodies against IL-36α, IL-36β, IL-36γ, and IL-36 receptor (IL-36R). Secondary antibodies conjugated to HRP were used for detection with DAB as the chromogen. Slides were counterstained with hematoxylin. Staining intensity and distribution were evaluated semi-quantitatively by two independent pathologists. Representative images were captured using a Leica microscope.

IHC results were semi-quantitatively evaluated by two independent pathologists blinded to the clinical data. An immunoreactive score (IRS) was calculated by multiplying the staining intensity and the proportion of positively stained cells. Staining intensity was scored as follows: 0 (no staining), 1 (weak), 2 (moderate), and 3 (strong). The proportion of positive cells was scored as: 0 (0%), 1 (1–10%), 2 (11–50%), 3 (51–80%), and 4 (>80%). The final IRS ranged from 0 to 12.

IHC scores were compared among the three groups (Normal, Pancreatitis, and PDAC) using the Kruskal–Wallis test. Subsequently, pairwise comparisons were performed between the Normal group and each of the other two groups (Pancreatitis and PDAC) using Dunn's post hoc test with Bonferroni correction for multiple comparisons. A two-tailed P value < 0.05 was considered statistically significant. Statistical analyses were performed using GraphPad Prism version 8.4.

Correlation analysis using TCGA pancreatic cancer data

Transcriptomic data for pancreatic adenocarcinoma (PAAD) were obtained from The Cancer Genome Atlas (TCGA) via the Genomic Data Commons (GDC) portal (https://portal.gdc.cancer.gov/). RNA-seq data were downloaded in transcripts per million (TPM) format and transformed as $\log_2(\mathrm{TPM} + 1)$ for downstream analysis.

To investigate the potential biological functions of IL1RL2 (IL-36R), we assessed its association with tumor-related gene expression programs, including inflammatory response, TGF-β signaling, tumor inflammation signature, collagen formation, extracellular matrix (ECM) degradation, and epithelial–mesenchymal transition (EMT).

Signature scores were calculated using the single-sample Gene Set Enrichment Analysis (ssGSEA) method implemented in the GSVA package (version 1.44.5) in R (version 4.2.2). Gene sets were curated from the MSigDB v7.5.1 database and published literature.

Spearman correlation analysis was performed between IL1RL2 expression and each signature score across 179 PAAD tumor samples. Correlation coefficients (ρ), p-values, and 95% confidence intervals (95% CI) were calculated using the cor.test() function. Scatter plots with fitted trend lines (loess) and 95% CI shading were generated using ggplot2 (version 3.4.0), and marginal density plots were added using the ggExtra package (version 0.10.0). All statistical analyses and visualizations were performed in RStudio (version 2022.12.0).

Animal experiments

Subcutaneous pancreatic tumor model and IL-36RA treatment

Animal experiments were conducted in accordance with institutional guidelines and approved by the Animal Ethics Committee of Qingyuan People's Hospital (Approval No. LEAC-2023-013). Six-week-old female nude mice were subcutaneously inoculated in the right flank with 1×10^6 human pancreatic cancer cells (CFPAC-1), while C57BL/6 mice were similarly injected with 1×10^6 syngeneic murine pancreatic cancer cells (Panc02). Mice were randomized into control and treatment groups (n = 5 per group) when tumors reached approximately 100 mm^3 for CFPAC-1 xenografts and 50 mm^3 for Panc02 xenografts. For Panc02 tumors, a smaller baseline volume was selected because this syngeneic model exhibits more aggressive growth in immunocompetent mice. This strategy ensured an adequate therapeutic window and minimized the risk of animal loss due to rapid tumor progression. The treatment group received intraperitoneal injections of recombinant IL-36 receptor antagonist (IL-36RA, 100 µg/mouse, three times per week) for 3 weeks. Tumor volumes were measured every 3 days using calipers and calculated using the formula: $(\text{length} \times \text{width}^2)/2$. At the endpoint, tumors were excised, weighed, photographed, and subjected to further histological and molecular analyses.

Animal model of acute pancreatitis and IL-36RA treatment

Acute pancreatitis was induced in mice by repeated intraperitoneal injections of cerulein (Sigma-Aldrich; 50 µg/kg body weight) administered hourly for 6 consecutive hours, as previously described. The control group (Cerulein) received cerulein alone, while the treatment

group (IL-36RA) received cerulein in combination with recombinant mouse IL-36 receptor antagonist (IL-36RA, Saloarbio; 100μg per mouse) administered intraperitoneally 30 minutes prior to the first cerulein injection and again 3 hours later. Pancreatic tissues were collected at 3, 12, 36, 60, 84, and 96 h after the first injection to represent both the inflammatory (3–36 h) and regenerative (60–96 h) phases. Tissues were fixed in 4% paraformaldehyde, embedded in paraffin, sectioned at 5 μm, and stained with hematoxylin and eosin (H&E) for histological analysis under light microscopy.

Histological analysis

Excised tumor tissues were fixed in 10% formalin, embedded in paraffin, sectioned at 4 μm, and stained with hematoxylin and eosin (H&E). Histological evaluation was performed under light microscopy. Apoptotic features including nuclear pyknosis, fragmentation, and cytoplasmic condensation were recorded.

Western blotting analysis

Subcutaneous xenograft tumor tissues derived from CFPAC-1 or Panc02 cells were homogenized in RIPA buffer (Thermo Fisher, A32959) containing protease and phosphatase inhibitor cocktail on ice for 30 min. Lysates were centrifuged at 14,000 × g for 30 min at 4°C to remove debris, and protein concentrations were determined using the BCA protein assay kit (Beyotime, P0010). Equal amounts of protein (30 μg per lane) were mixed with 5× Laemmli sample buffer containing 2-mercaptoethanol, denatured at 100 °C for 5 min, and separated on 10% SDS–PAGE gels. Proteins were transferred onto PVDF membranes (Millipore) at 100 V for 1 h using a wet transfer system. Membranes were blocked in 5% non-fat milk prepared in TBST (20 mM Tris-HCl, 150 mM NaCl, 0.1% Tween-20) for 1 h at room temperature, followed by overnight incubation at 4°C with primary antibodies against Bcl-2 (ABclonal, A19693; 1:1000), Bax (ABclonal, A19684; 1:1000), and GAPDH (ABclonal, AC002; 1:10000). After washing, membranes were incubated with DyLight 800-conjugated goat anti-rabbit IgG (H+L) (Thermo Fisher, SA5-10036; 1:5000) and DyLight 680-conjugated goat anti-mouse IgG (H+L, cross-adsorbed) (Thermo Fisher, 35519; 1:5000) for 1 h at room temperature. Fluorescent signals were detected using the Odyssey Fc imaging system (LI-COR Biosciences), with DyLight 800 displayed in green and DyLight 680 in red. Band intensities were quantified by densitometry using ImageJ software and normalized to GAPDH.

CCK-8 cell viability assay

Cell viability was measured using the Cell Counting Kit-8 (Beyotime, C0038). CFPAC-1 and Panc02 cells were seeded into 96-well plates at a density of 4×10^3 cells per well and cultured

overnight. Cells were then treated with recombinant IL-36RA at the indicated concentrations, or pre-stimulated with recombinant IL-36γ followed by IL-36RA treatment. After 24 h, 10 μL of CCK-8 solution was added to each well and incubated at 37°C for 1 h. Absorbance was measured at 450 nm using a microplate reader. Relative cell viability was calculated by normalizing to vehicle controls, and the data were presented as mean ± SEM from at least three independent experiments.

TUNEL assay

Apoptosis in tissue sections was evaluated using the One-Step TUNEL Apoptosis Assay Kit (Beyotime, C1090). Paraffin-embedded sections were deparaffinized, rehydrated, and permeabilized according to the manufacturer's instructions. Sections were then incubated with TUNEL detection solution containing TdT and Cy3-dUTP at 37°C for 60 min in the dark. After PBS washes, nuclei were counterstained with DAPI, and sections were mounted with antifade medium. TUNEL-positive apoptotic cells were visualized using the Cy3 channel and quantified as the percentage of positive cells per field.

Three-color immunofluorescence staining

Three-color immunofluorescence was performed on 4μm paraffin-embedded pancreatic tissue sections. After deparaffinization and rehydration, antigen retrieval was carried out using citrate buffer (pH 6.0), followed by blocking with 5% bovine serum albumin for 30 min at room temperature. Sections were incubated overnight at 4°C with two primary antibodies derived from different host species. After PBS washes, corresponding fluorescence-conjugated secondary antibodies were applied for 1 h at room temperature in the dark. Nuclei were counterstained with DAPI using an antifade mounting medium. Fluorescence images were acquired using identical exposure settings, and negative controls were prepared by omitting primary antibodies.

Multiplex immunofluorescence staining

Multiplex immunofluorescence was performed using SignalStar™ Multiplex IHC Kits & Reagents (Cell Signaling Technology) according to the manufacturer's protocol. Oligo-conjugated primary antibodies against Pan-Keratin (CST, 30754), CD3 (CST, 47856), CD8 (CST, 39714), and F4/80 (CST, 95804) were applied to 4μm FFPE pancreatic tissue sections at 1:50 dilution. After hybridization and signal amplification steps provided by the SignalStar™ workflow, fluorescence signals were detected in multiple channels. Nuclei were counterstained with DAPI using antifade mounting medium. Fluorescence images were acquired with a confocal microscope under identical exposure settings, and negative controls were prepared

by omitting primary antibodies.

Statistical analysis

All data are presented as mean ± SEM. Comparisons between groups were performed using unpaired Student's t-test. A p-value of <0.05 was considered statistically significant. GraphPad Prism 9.0 was used for data visualization and statistical analysis.

2. Supplementary Tables

Supplementary Table S1. Reagents and Antibodies

Reagent	Company	Catalog No.	Application
Recombinant Murine IL-36 Receptor Antagonist	Beyotime Biotechnology	P5993	*In vivo* and *in vitro* assay
Recombinant Human IL-36 Receptor Antagonist	Beyotime Biotechnology	P5273	*In vivo* and *in vitro* assay
Recombinant Human IL-36γ, 169a.a.	Beyotime Biotechnology	P5264	*In vitro* assay
Recombinant Murine IL-36γ, 152a.a.	Beyotime Biotechnology	P5991	*In vitro* assay
IL1F9 antibody	ThermoFisher	PA599822	IHC (1:100)
IL1F6 antibody	ThermoFisher	PA5100832	IHC (1:100)
IL36β antibody	ThermoFisher	PA5100833	IHC (1:100)
Anti-IL1RL2 antibody	Solarbio Life Science	K001842P	IHC (1:100); IF (1:100)
Cerulein	MCE	HY-A0190	*In vivo*
Bcl-2	ABclonal	A19693	WB (1:1000)
Bax	ABclonal	A19684	WB (1:1000)
GAPDH	ABclonal	AC002	WB (1:10000)
DyLight 800-conjugated goat anti-rabbit IgG (H+L)	ThermoFisher	SA5-10036	WB (1:5000)
DyLight 680-conjugated goat anti-mouse IgG (H+L, cross-adsorbed)	ThermoFisher	35519	WB (1:5000)
Pan-Keratin	Cell Signal Technology	30754	IF (1:50)
CD3	Cell Signal Technology	47865	IF (1:50)
CD8	Cell Signal Technology	39714	IF (1:50)
F4/80	Cell Signal Technology	95804	IF (1:50)
PD-L1	Beyotime Biotechnology	AF7710	IHC (1:100)
TUNEL Kit	Beyotime Biotechnology	C1090	Apoptosis detection
CCK8	Beyotime Biotechnology	C0038	Cell viability assay

3. Supplementary Figures

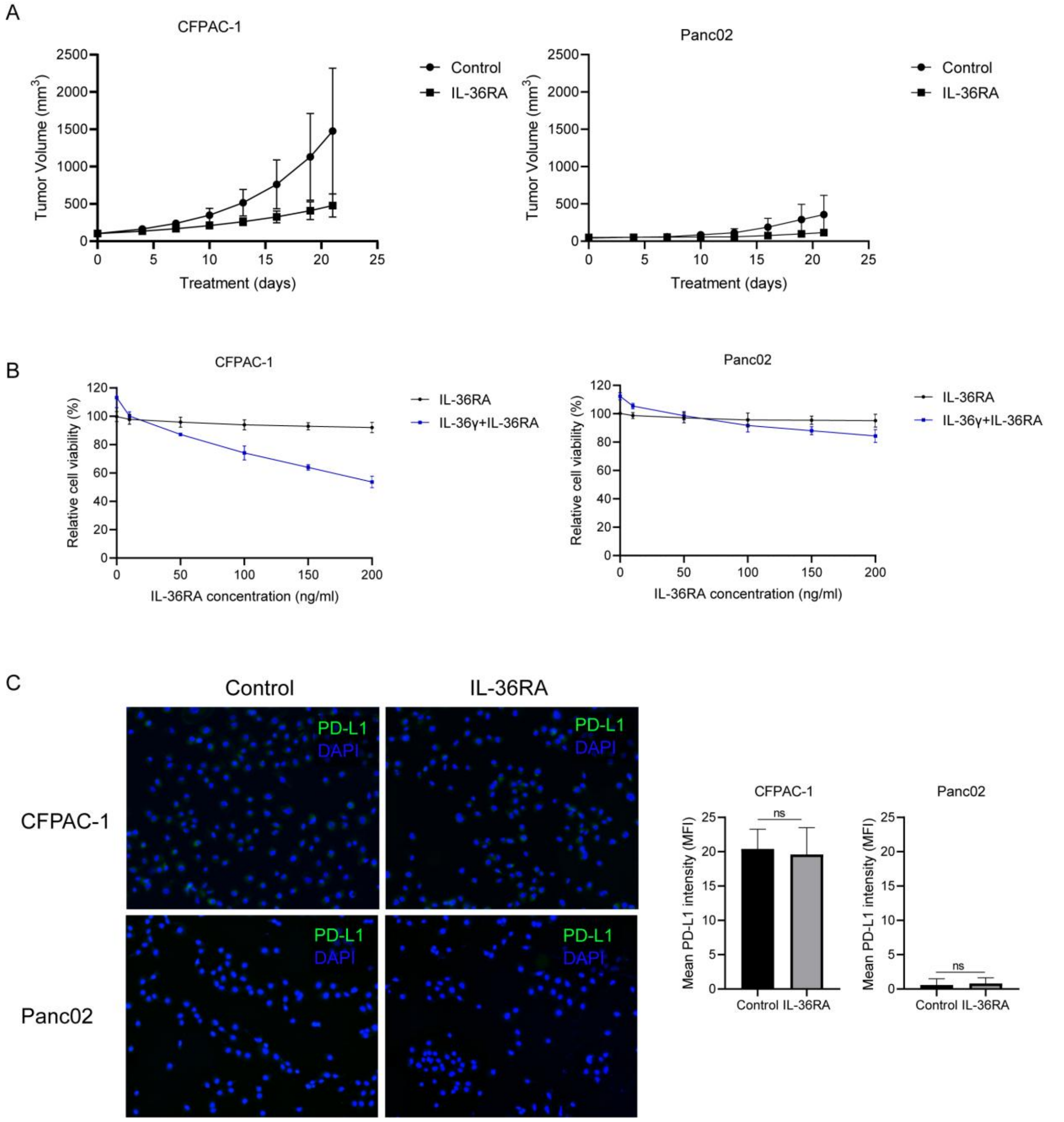

Fig. S1. Effects of IL-36RA on tumor growth, cell viability, and PD-L1 expression in pancreatic cancer models. (**A**) Growth curves of CFPAC-1 xenografts in nude mice and Panc02 tumors in C57BL/6 mice injected intraperitoneally with IL-36RA (100 μg/mouse, three times per week) or vehicle control. Tumor volumes are measured every 3 days and shown as mean ± SD (n = 5 per group). (**B**) CCK-8 assays showing relative viability of CFPAC-1 and Panc02 cells treated with increasing concentrations of IL-36RA, with or without IL-36γ stimulation. Data are presented as mean ± SEM from three independent experiments. (**C**) IF staining of PD-L1 (green) and nuclei (DAPI, blue) in CFPAC-1 and Panc02 cells treated with vehicle or IL-36RA. Quantification of mean fluorescence intensity (MFI) of PD-L1 is shown on the right. Data are expressed as mean ± SEM; ns, not significant.

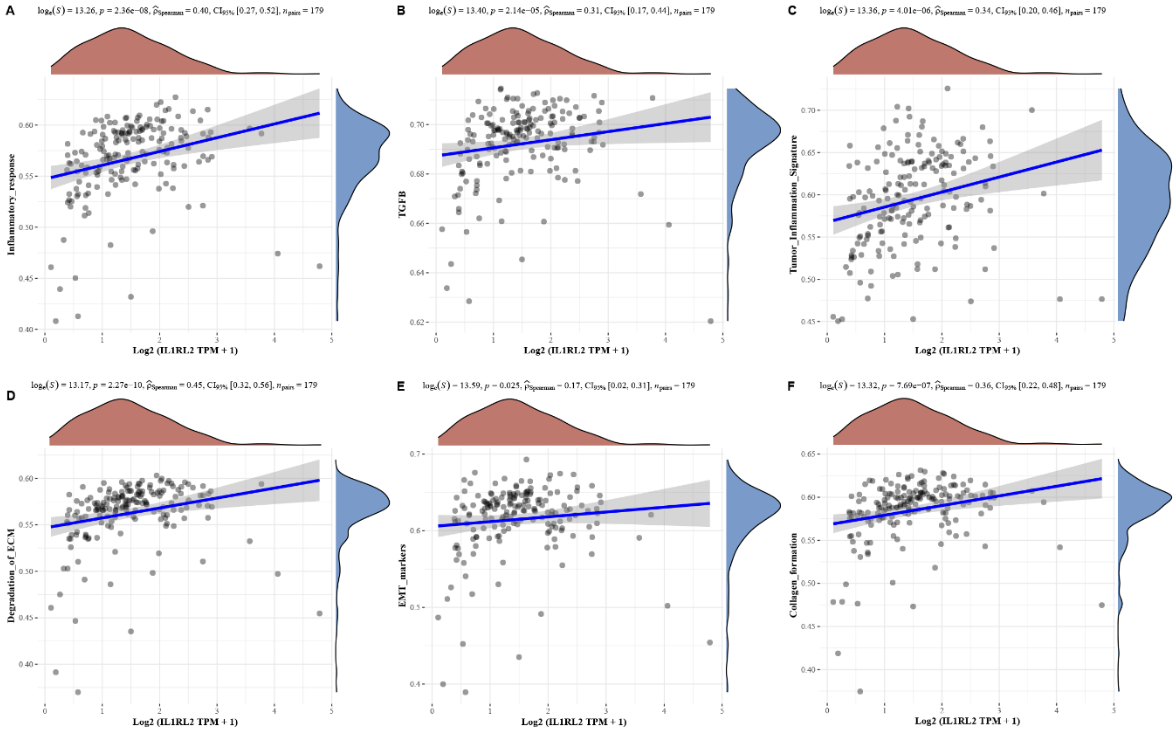

Fig. S2. Correlation of IL1RL2 (IL-36R) expression with tumor-related gene signatures in TCGA pancreatic adenocarcinoma. (**A**–**F**) Spearman correlation analysis between IL1RL2 (IL-36R) mRNA expression [$\log_2$(TPM + 1)] and tumor-related gene expression signatures in pancreatic adenocarcinoma (PAAD) samples from The Cancer Genome Atlas (TCGA) (n=179). IL1RL2 expression showed significant positive correlations with inflammatory response (**A**), TGF-β signaling (**B**), tumor inflammation signature (**C**), collagen formation (**D**), degradation of extracellular matrix (ECM) (**E**), and epithelial–mesenchymal transition (EMT) markers (**F**). Each dot represents a tumor sample. Spearman correlation coefficient (ρ), p-value, and 95% confidence interval (CI) are displayed in each panel. Density plots on the margins indicate the distribution of IL1RL2 expression (x-axis) and the corresponding signature score (y-axis). Trend lines represent smoothed fit with 95% CI shading for visualization purposes only.

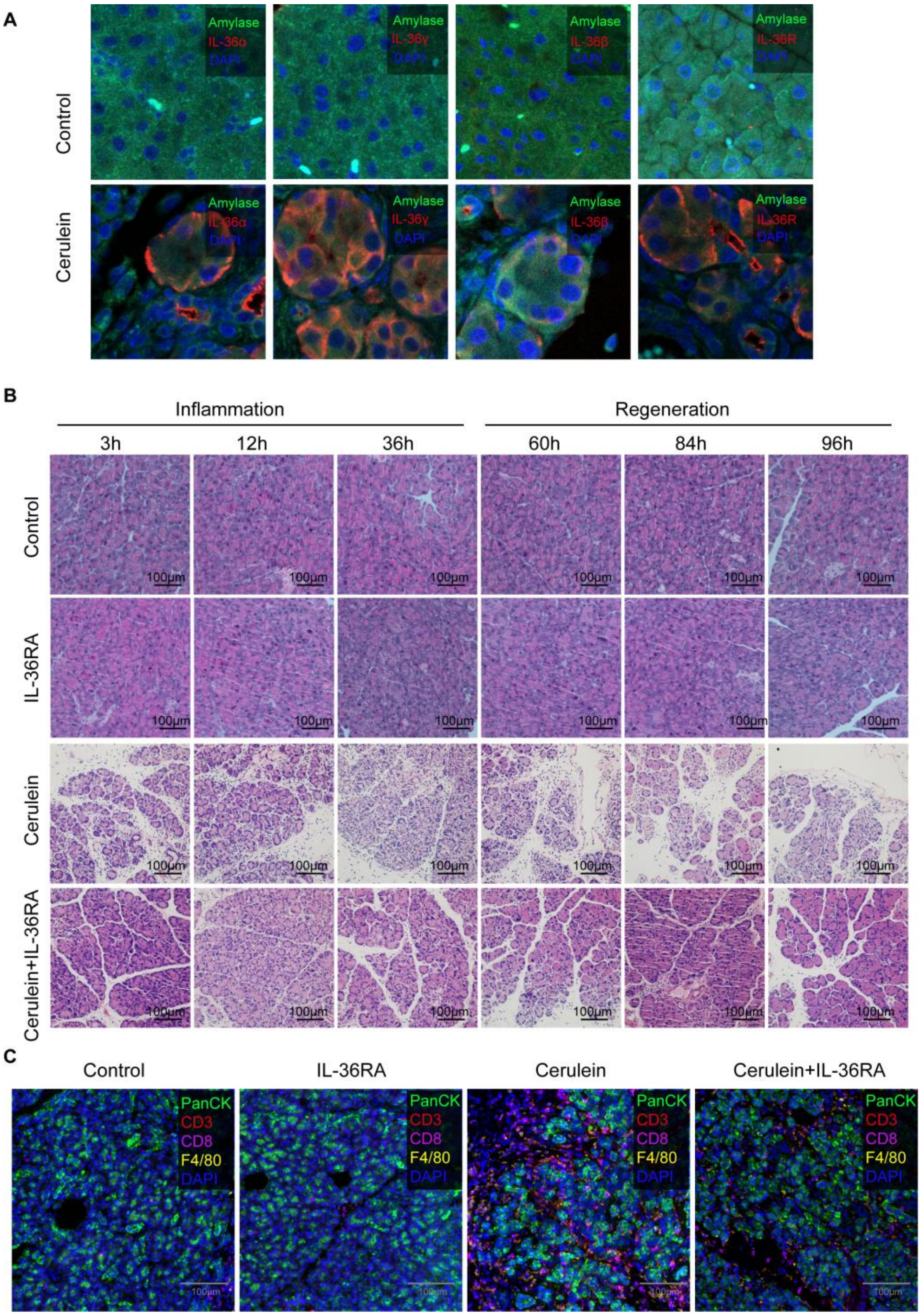

Fig. S3. IL-36 expression and the effects of IL-36RA in cerulein-induced acute pancreatitis. (**A**) Representative immunofluorescence staining of amylase (green), IL-36α, IL-36β, IL-36γ, and IL-36R (red) in pancreatic tissues from control and cerulein-treated mice. Nuclei were counterstained with DAPI (blue). (**B**) Representative H&E staining of pancreatic tissues from control, IL-36RA, cerulein, and cerulein+IL-36RA groups at indicated time points during inflammation (3, 12, 36 h) and regeneration (60, 84, 96 h). Cerulein induced extensive acinar cell damage and inflammatory infiltration, which were markedly attenuated by IL-36RA treatment. Scale bar, 100 μm. (**C**) Representative multiplex immunofluorescence staining of pancreatic tissues at 36 h showing PanCK (green), CD3 (red), CD8 (purple), F4/80 (orange), and DAPI (blue) in control, IL-36RA, cerulein, and cerulein+IL-36RA groups.
